# Hemp Essential Oils as Novel Antioxidant and Bacteriostatic Agents in PLA-Based Packaging

**DOI:** 10.3390/polym18070824

**Published:** 2026-03-27

**Authors:** Eugenia Mazzara, Annafelicia Civitavecchia, Pierluigi Stipa, Cristina Minnelli, Emiliano Laudadio, Tiziano Bellezze, Pietro Forcellese, Samuele Rinaldi, Kateryna Fatyeyeva, Gianluca Morroni, Gloria D’Achille, Simona Sabbatini, Francesca Luzi

**Affiliations:** 1 Food Chemistry and Technology Department, Teagasc Food Research Centre, Ashtown, D15 DY05 Dublin, Ireland; eugenia.mazzara@teagasc.ie; 2 Department of Science and Engineering of Matter, Environment and Urban Planning, Università Politecnica delle Marche, Via Brecce Bianche, 60131 Ancona, Italy; a.civitavecchia@staff.univpm.it (A.C.); p.stipa@staff.univpm.it (P.S.); e.laudadio@staff.univpm.it (E.L.); t.bellezze@staff.univpm.it (T.B.); 3 Department of Life and Environmental Sciences, Università Politecnica delle Marche, Via Brecce Bianche, 60131 Ancona, Italy; c.minnelli@staff.univpm.it (C.M.); s.rinaldi@staff.univpm.it (S.R.); 4 Department of Theoretical and Applied Sciences, eCampus University, Via Isimbardi, 10, 22060 Novedrate, Italy; pietro.forcellese@uniecampus.it; 5 Normandie University, UNIROUEN, INSA ROUEN, CNRS, Polymères Biopolymères Surfaces (PBS), 76000 Rouen, France; kateryna.fatyeyeva@univ-rouen.fr; 6 Departements of Biomedical Sciences and Public Health, Università Politecnica delle Marche, Via Tronto 10/A, 60126 Ancona, Italy; g.morroni@staff.univpm.it (G.M.); g.dachille@staff.univpm.it (G.D.); 7 Microbiology Laboratory, Azienda Ospedaliero Universitaria delle Marche, Via Conca 71, 60126 Ancona, Italy

**Keywords:** hemp essential oils, polylactic acid, water permeation, antioxidant properties

## Abstract

Poly(lactic acid) (PLA) films containing two different hemp-derived essential oils (EOs), Carmagnola CS (Carm) and Futura 75 (Fut), at 1, 5, and 10% wt were successfully produced via solvent casting for packaging applications. The influence of EO presence, type, and concentration on the chemical, morphological, and thermal properties of the PLA-based films was investigated. In addition, radical-scavenging activity, water transport properties, and antimicrobial performance were evaluated to assess the effect of EOs on the structural and functional characteristics of the resulting packaging materials. FTIR spectroscopy confirmed the successful incorporation of the hemp essential oils Carm and Fut into the polymer matrix, with a concentration-dependent effect that is more pronounced for Fut than for Carm. In the second heating run, evaluated by DSC measurements, both EOs lowered T_g_ from 60.3 °C (PLA) to 52.0 °C for PLA_10 Carm and 55.1 °C for PLA_10 Fut. The EOs act as plasticizers in the PLA matrix, improving the deformation at break. Gas barrier measurements showed that permeability decreased from 3027 ± 300 Barrer (PLA) to (2499 ± 44) Barrer in PLA_10 Carm and 2623 ± 130 Barrer in PLA_10 Fut, with a corresponding reduction in diffusivity. The barrier improvement factor reached 17% for Carm and 15% for Fut, confirming the enhanced barrier performance of PLA_EOs films. DPPH assays showed that PLA_EOs films retained most of the antioxidant activity of the free oils, with only a 10–15% reduction for PLA_Fut and no significant loss for PLA_Carm after one week. After one month, the activity of Carm in PLA film decreased by 18%, whereas the performance of its free form remained unchanged, confirming the superior and more stable radical scavenging capacity of Carm compared to Fut. Overall, the study demonstrates that hemp essential oils can be effectively integrated into PLA without compromising structural integrity, while preserving antioxidant performance and enhancing water barrier properties, supporting their potential as sustainable active packaging components.

## 1. Introduction

Plastics have become indispensable to modern technological progress, yet their widespread use has triggered profound environmental challenges that increasingly overshadow their benefits. The term “Plastic Age” not only reflects their ubiquity but also underscores the ecological crisis they contribute to [1]. This growing concern has spurred demand for eco-friendly alternatives, driving extensive research into more sustainable polymers capable of combining high performance with cost efficiency [2]. As a result, numerous polymers, as well as the corresponding composites derived from renewable sources, are being developed to meet sustainability goals [3].

In recent years, the packaging sector has undergone a paradigm shift toward sustainability, driven by environmental concerns, regulatory pressures, and consumer demand for safer materials. Although effective, conventional petroleum-based plastics pose ecological and health risks owing to their accumulation and the potential leaching of harmful additives. This has led to increased focus on packaging films derived from renewable resources, which offer reduced environmental impact and improved safety profiles [4].

**Figure 1:**
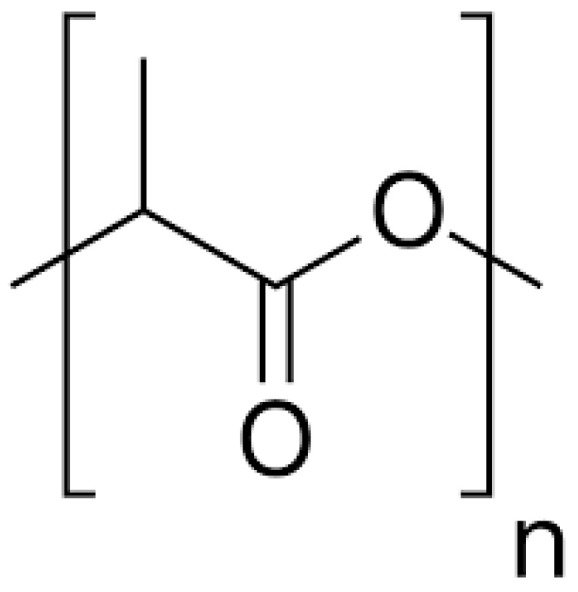
Chemical structure of PLA.

PLA (Figure 1), classified as “Generally Recognized as Safe” (GRAS) by regulatory authorities, has gained traction in food packaging and other sectors such as biomedical devices, agriculture, textiles, and additive manufacturing [5,6]. This polymer offers several advantages, primarily due to its renewable, bio-based origin, which reduces dependence on fossil resources and can lower the overall carbon footprint compared with conventional plastics [7,8]. PLA exhibits good transparency and gloss, making it attractive for food and consumer packaging, and provides relatively high stiffness and strength, enabling the production of rigid containers and films with good dimensional stability [9]. It can be processed using standard polymer technologies such as extrusion, thermoforming, and injection moulding, facilitating industrial adoption. In addition, PLA is industrially compostable, which supports circular-economy strategies when appropriate waste-management infrastructures are available [10]. Nonetheless, its relatively low toughness, poor thermal resistance, and limited barrier properties compared to conventional plastics have prompted the development of active packaging strategies to enhance its functionality [11].

Since PLA films could also be used for food packaging, they should be endowed with antibacterial properties or, at least, should not favour bacterial growth. For this reason, the use of natural products has gained increasing interest over the last decade. Therefore, the design of functional antibacterial materials derived from biomass remains a significant challenge for the scientific community.

A promising approach involves incorporating essential oils (EOs), volatile and lipophilic compounds extracted from aromatic plants, into PLA matrices. EOs are rich in terpenes and phenolic compounds, which exhibit potent antioxidant, antimicrobial, antifungal, and insecticidal activities [12]. Their integration into polymer films has been shown to affect mechanical strength, UV resistance, and microbial inhibition, as demonstrated with EOs from bergamot, lemongrass, rosemary, thyme, and clove [13,14]. Essential oils are generally recognized as safe (GRAS) and approved by the U.S. Food and Drug Administration (FDA) [15], facilitating their use in food packaging systems [16]. Among the various constituents of essential oils, oxygenated terpenoids, aldehydes, and phenols stand out as the most potent antimicrobial agents [17]. Thyme [18], cinnamon [19], rosemary [20], clove [21], etc., are among the most used plants to obtain natural extracts for application in antimicrobial food packaging [22]. Muñoz-Gimena and co-authors proposed the combination of cinnamon essential oils in starch and seed flour-based material, obtained by the revalorization of avocado seeds, to develop active packaging systems [23]. Mohammedi and Fasihi proposed the development of an eco-sustainable packaging system based on PLA and thermoplastic starch (TPS), modified by combining clove essential oil (CEO) and cochineal dye to simultaneously monitor spoilage and preserve high-protein foods [24]. The inclusion of CEO enhanced antibacterial effectiveness against *Listeria monocytogenes* and *Escherichia coli*. The active film successfully extended the shelf life of food products providing a visible spoilage indicator through color modification. Therefore, the films incorporating essential oils could serve as a protective barrier for contaminating microorganisms on the surface of food and reduce lipid oxidation, thus improving the quality and safety of coated products. Essential oils are regarded as natural alternatives to chemical preservatives and meet the demands of consumers for mildly processed or natural products in the use of food packaging [25].

An important parameter for EOs incorporation into PLA matrices is the polymer solubility, that varies according to crystallinity and molecular weight. PLA used in this work is soluble only in chloroform and dichloromethane (DCM), thus preventing the use of non-toxic and environmentally benign solvents (e.g., ethyl acetate). In addition, preliminary experiments showed irregular film formation with the less toxic DCM, thus the use of chloroform was mandatory. However, the current stringent environmental regulations (e.g., chloroform releases ≤ 50 ppm in many countries) have recently led to the construction of new plants or the upgrade of existing equipment, so that many large manufacturers now exploit the very efficient strategies available nowadays for the recovery and reuse of chloroform on industrial scale, when its use is unavoidable.

Despite extensive research on plant-derived EOs, hemp essential oils (HEOs) remain largely unexplored in the context of PLA-based packaging. Industrial hemp, particularly cultivars like Fut and Carm, produces EOs rich in monoterpenes (e.g., α-pinene, β-myrcene, terpinolene) and sesquiterpenes (e.g., β-caryophyllene, α-humulene, caryophyllene oxide) from glandular trichomes on its inflorescences, often discarded as agro-industrial waste [12,26]. These compounds have demonstrated selective antimicrobial activity, especially against Gram-positive bacteria such as *Staphylococcus aureus* and *Enterococcus* spp., with minimum inhibitory concentrations (MICs) varying by cultivar and strain [27,28].

Moreover, rising concerns over the toxicity of synthetic antioxidants like butylated hydroxytoluene (BHT) and butylated hydroxyanisole (BHA) have intensified interest in natural alternatives. HEOs, particularly those from Fut, have shown superior radical scavenging and reducing power in antioxidant assays, positioning them as viable candidates for active packaging applications [29,30].

On this basis, considering the prevalence of Carm and Fut varieties at national and regional levels, the superior EOs yield, and the antimicrobial and antioxidant effects mainly associated with Fut EOs, these two varieties have been selected to conduct the current study [31,32]. To date, no studies have systematically investigated the incorporation of HEOs into PLA films. This research addresses that gap by evaluating the structural, thermal, antioxidant, and antimicrobial properties of PLA-HEO blend films produced via solvent casting. Characterization was performed using Fourier Transform Infrared (FTIR) spectroscopy for molecular interactions, Differential Scanning Calorimetry (DSC) and Thermogravimetric Analysis (TGA) for thermal behavior and the 1,1-diphenyl-2-picrylhydrazyl (DPPH) radical scavenging assay to evaluate antioxidant activity. Antimicrobial efficacy was assessed through the MIC determination of EOs, and adhesion assays, while microscopy examinations were employed to elucidate surface morphology and EO distribution within the polymer matrix.

This work aims to develop PLA-based composites incorporating hemp-derived bioactive compounds and to assess their suitability for packaging applications. The main objectives are to evaluate their mechanical, antimicrobial, barrier, and antioxidant properties, which remain scarcely investigated in the literature but may offer relevant functional advantages. The study also considers the potential of agro-industrial by products as sustainable sources of these bioactive components.

## 2. Materials and Methods

### 2.1. Plant Material

The monoecious inflorescences of Fut hemp variety and the female inflorescences of the dioecious Carm hemp variety were cultivated by La Biologica farm (Fiuminata, Italy) and harvested in September 2020. The plant material was then dried and submitted to EOs extraction.

### 2.2. Hemp EOs Extraction

Fut and Carm EOs were obtained by hydrodistillation (HD) as the most conventional technique for producing plant EOs. Briefly, two biomass samples belonging to the two hemp varieties (1500 g each) were placed in round bottomed flasks of 20 L capacity, filled with 12 L each of distilled water. The equipment was completed with a Falc MA heating mantle (Falc Instruments, Treviglio, Italy) and a Clevenger-type apparatus, in order to perform the distillation of the EOs for 4 h. The EOs were recovered apart from water and kept in amber glass vials at 4 °C until their use. The EOs yields were estimated on a dry weight basis (*w*/*w*).

### 2.3. Chemical Analysis of Hemp EOs

The chemical profile of hemp EOs was investigated by gas chromatography coupled to mass spectrometry (GC-MS) analysis. For the purpose, an Agilent 8890 GC, equipped with a single quadrupole Agilent 5977B Mass Spectrometer (MSD) (Santa Clara, CA, USA), and a PAL RTC 120 autosampler (CTC Analytics AG, Zwingen, Switzerland) was employed, using a non-polar HP-5MS (5% phenylmethylpolysiloxane; 30 mL, 0.25 mm i.d., 0.25 µm f.t.) capillary column with a helium (He) flow rate of 1 mL min^−1^ employing the temperatures ramps previously described [12]. EOs samples were diluted 1:100 in LC-MS grade *n*-hexane and injected in split-mode (1:200). The SCAN mode (40–400 *m*/*z*) was employed for data acquisition, further analyzed by means of the MSD ChemStation software (Agilent, Version G1701DA D.01.00), the NIST Mass Spectral Search Program v.2.4 for the NIST/EPA/NIH EI, and the NIST Tandem Mass Spectral Library v. 2.3. The linear retention indices (RI) were calculated by injecting a standard mix of *n*-alkanes (C_8_-C_30_, Supelco, Bellefonte, CA, USA) and compared with those found in ADAMS, FFNSC2, and NIST 17 commercial libraries [33]. Similarly, such correspondence was also checked for mass spectra (MS). Relative abundance (peak area percentages) was determined by normalizing without using correction factors.

### 2.4. Preparation of PLA_EOs Blend Films

Poly(lactic acid) (PLA) Ingeo 4043D (*L*-lactic acid/*D*-lactic acid ratios from 24:1 to 30:1, *d* = 1.24 g cm^−3^, T_m_ = 145–160 °C) was purchased from NatureWorks LCC (Minnetonka, MN, USA). The number average molecular weight (M_n_) of 106 kDa and dispersity (Đ) of 1.84 were measured by gel permeation chromatography, using a Shimadzu NEXERA GPC (Shimadzu corporation, Kyoto, Japan) system equipped with a refractive index detector, and using THF as the eluent and polystyrene standards for the calibration.

PLA and PLA_EOs films were prepared by the solvent-casting method by using the procedure already described [34].

The content of the EOs was selected based on preliminary experiments which showed proper phase blending and films integrity up to and not above 10%, and according to previous studies reporting antimicrobial and antioxidant effects at the same concentrations of other plant EOs loaded into biodegradable films [35,36].

First, PLA (1 g) was dissolved in 20 mL of chloroform (CHCl_3_) with stirring at room temperature (RT). The obtained solution was cast onto a Petri dish and then dried for 24 h at RT (24 ± 2 °C).

Active PLA_EO-based films were obtained by mixing, under magnetic stirring, the polymer solution with a specific amount of EO (1, 5 and 10% wt EO) for 1 h at RT. This allowed obtaining a homogeneous dispersion of the active additive.

The polymer solution was then cast onto a Teflon^®^ substrate (15 cm of diameter Petri dish) and dried at RT. The resulting PLA films were carefully peeled off and stored in a desiccator (24 °C, 33% RH) until investigations. PLA films with an average thickness of 90 ± 10 µm as measured by a digital micrometer Mitutoyo at 10 different locations uniformly distributed over the film surface were obtained.

### 2.5. ATR-FTIR Measurements of EOs and Films

IR spectra were acquired in reflectance mode using a PerkinElmer SpectrumGX1 spectrometer (Waltham, MA, USA) equipped with a ZnSe crystal ATR accessory. The spectral range was 4000–500 cm^−1^, with a spectral resolution of 4 cm^−1^; each spectrum was the result of 32 scans. Samples were placed directly onto the ZnSe crystal without any preparation. To evaluate the homogeneity of each film, five IR spectra were acquired at five different points of each sample. The average absorbance spectra were calculated; the background spectrum was collected on the clean crystal under the same conditions. Raw IR spectra were converted to absorbance mode and vector normalized (Spectrum10.4.0 software, PerkinElmer, USA).

### 2.6. TGA of EOs and Films

The TGA of hemp EOs, pure PLA film, and PLA_EOs films was carried out using a Mettler Toledo TGA/SDTA 851 (Columbus, OH, USA) by heating the materials from 25 °C up to 900 °C at 10 °C min^−1^ under nitrogen flow (60 mL min^–1^).

### 2.7. DSC of Films

DSC tests were carried out with a Seiko EXSTAR 6000 calorimeter (Tokio, Japan), performing two heating and one cooling scans from −25 °C to 210 °C, at 10 °C min^−1^ under a nitrogen flow rate of 50 mL min^−1^. Glass transition (T_g_), cold crystallization and melting temperatures (T_cc_ and T_m_) and enthalpies (ΔH_cc_ and ΔH_m_) for pure PLA film and the different PLA_EOs films were determined during first and second heating scans. The analysis was carried out in triplicate, and the results were reported as the mean value ± standard deviation.

The crystallinity degree was calculated as (Equation (1)):

(1) $$X=\frac{1}{\left(1\;-\;m_{f}\right)}\frac{{\Delta H}_{m}-{\Delta H}_{cc}}{{\Delta H}_{0}}*100$$

where ΔH is the enthalpy for melting (ΔH_m_) or cold crystallization (ΔH_cc_), ΔH_0_ is the enthalpy of melting for a 100% crystalline PLA sample, taken as 93 J g^−1^ and (1 − m_f_) is the weight fraction of PLA in the sample [37].

### 2.8. Microscopy Examinations

The morphology of the surface of the PLA and PLA_EOs films was investigated by means of an Olympus BX51 optical microscope (Shinjuku, Tokyo, Japan). The images were taken to evaluate the EOs distribution on the surface of the different blends of PLA_EOs films and for comparison with the morphology of the PLA film.

The microstructure of the PLA and PLA_EOs films was investigated trough cryo-fractured cross-sectional surfaces using a Zeiss Supra 40-VP (Germany) high vacuum Field Emission Scanning Electron Microscopy (FE-SEM) after gold sputtering and by using an accelerating voltage of 3 kV.

### 2.9. Mechanical Characterization

Tensile properties of PLA and PLA_EOs films have been measured according to the ISO 527-3 standard [38] method. Strips of 150 mm × 25 mm were cut from each film type. The strips were mounted on an ACQUATI AG/MC dynamometer for the tensile tests. The elastic modulus (E_Young_), tensile strength (σ_b_) and elongation (ε_b_) at break were determined.

### 2.10. Transport Properties

The water permeation measurements were performed with a home-built apparatus at 25 ± 1 °C [39]. The permeameter consists of a measurement cell, a dry nitrogen supply and a chilled mirror hygrometer (General Eastern Instruments, Billerica, MA, USA). The film with a *L* thickness was mounted in the cell and dry nitrogen was being flushed into both compartments (downstream and upstream) for hours until a dew point lower than -72 °C was obtained. Then, a stream of pure water (Milli-Q water system, resistivity 18 MΩ·cm^−1^) was pumped through the upstream compartment and the transfer of water through the film, i.e., from the upstream compartment to the downstream one, was monitored by the humidity sensor. For permeation measurement, the initial and boundary conditions are defined as Equations (2) and (3)):

*C* (*x*, *t*) = 0, 0 < *x* < *L* at *t* = 0 (2)

*C* (*0*, *t*) = *C_eq_* (constant) and *C* (*L*, *t*) = 0 at *t* > 0 (3)

The presented results were averaged out of at least three measurements performed for each film composition and the results are reported as a mean value ± standard deviation.

### 2.11. DPPH Assays

The DPPH radical scavenging assay was conducted to evaluate the antioxidant activity of PLA films with and without EOs. To prepare the assay, two stock solutions were made by incubating PLA films containing methanolic solutions of either Carm or Fut EOs at a concentration of 3.125 mg mL^−1^ and 1.563 mg mL^−1^ respectively for 24 h. On the following day, aliquots were mixed with an equal volume of DPPH solution (50 µg mL^−1^ in methanol) to achieve a final EO concentration of 1.563 mg mL^−1^ and 0.781 mg mL^−1^, respectively, in the reaction mixture. The mixtures were shaken thoroughly and incubated in the dark for 30 min. Absorbance measurements at 520 nm were taken using a BioTek Synergy HT MicroPlate Reader Spectrophotometer (Winooski, Vermont (USA), with PLA films serving as the blank and a DPPH methanol solution as the negative control. The percentage inhibition of the DPPH radical was calculated using the formula:

Inhibition ratio (%) = [(A_control_ − A_sample_)/A_control_] × 100 (4)

where A_control_ is the absorbance of the control solution containing DPPH and methanol (without EO), and A_sample_ is the absorbance of the reaction mixture containing the PLA films with or without EOs after incubation. All experiments were performed in triplicate, and each sample was tested at least three times to ensure reproducibility.

A calibration curve for each essential oil (EO) was established by preparing two stock solutions in methanol at concentrations of 15 mg mL^−1^ and 25 mg mL^−1^. These solutions were incubated under the same conditions used for PLA_EOs, as previously described, to ensure consistency (24 h). Serial dilutions were then performed, and aliquots from these dilutions were mixed with DPPH solution (50 µg mL^−1^ in methanol) to obtain final concentrations ranging from 12.5 mg mL^−1^ to 0.469 mg mL^−1^. The mixtures were shaken and incubated in the dark at room temperature for 30 min. Absorbance was measured at 520 nm using a BioTek Synergy HT MicroPlate Reader (Winooski, Vermont (USA), with methanol as the blank and DPPH solution as the negative control. Percent inhibition was calculated using Equation (4). The resulting calibration curves for both EOs exhibited a strong linear relationship, with R^2^ values of 0.98, demonstrating the method’s high reliability for quantifying the antioxidant activity of the EOs.

### 2.12. Kinetic Releases of EOs from PLA Films

Total immersion kinetic release tests were performed with 4 cm^2^ of PLA_EO 10% and 8 mL of release medium. The release tests of free EOs were also carried out using a dialysis method as previously described, with minor modifications. EOs were placed into dialysis bags (12,000 MW cut off) and dialysed against 8 mL of release medium as for the PLA_EOs.

The amount of EOs released into selected mediums was analyzed in terms of radical scavenging ability, using the stable radical DPPH method as previously described [40]. An aliquot of 0.5 mL of the release medium (water/ethanol 1:4, *v*/*v*) was taken at specific time intervals. The same volume of fresh 80% ethanol in Milli-Q water (water/ethanol 1:4, *v*/*v*) was then added to maintain a constant release volume. Then, each aliquot was mixed with 0.5 mL of a methanolic solution of DPPH (50 mg L^−1^) in a capped tube. The mixtures were shaken and incubated in the dark at room temperature for 30 min. The cumulative amount of EOs released was determined from the absorbance measured at 520 nm using a BioTek Synergy HT MicroPlate Reader (Winooski, Vermont (USA), with ethanol as the blank and DPPH solution as the negative control. Then, the DPPH percentage inhibition was calculated using Equation (4); the values shown in the graph correspond to the actual DPPH measurements obtained at each sampling time. All experiments were performed in triplicate, and each sample was tested at least three times to ensure reproducibility. The percentage released at each time point was expressed as a fraction of the total amount of EOs.

A calibration curve for each EO was established by preparing serial solutions in water/ethanol (1:4, *v*/*v*) to obtain final concentrations ranging from 1.56 mg mL^−1^ to 0.0976 mg mL^−1^. The resulting calibration curve exhibited a strong linear relationship, with an R^2^ value of 0.99 (Figure S4), demonstrating the method’s high reliability for quantifying the antioxidant activity of the EOs released.

To describe the EO release from the PLA matrix, the mathematical model developed by Korsmeyer–Peppas was applied. This semi-empirical model demonstrates a relation between the amount of drug released (essential oil, in this case) and time [41,42].

In particular, in this study the ratio of the amount of EO released at time *t*, *M_t_*, to the amount released at infinite time, *M_∞_*, is equal to the corresponding ratio of the inhibition observed at time *t*, *I_t_*, to that observed when all the EO has been released at infinite time, *I_∞_*, and according to the Korsmeyer–Peppas model is (Equation (5)):

(5) $$\frac{M_{t}}{M_{\infty }}=\frac{I_{t}}{I_{\infty }}=Kt^{n}$$

where *K* is the release rate constant and *n* is the release exponent and its value predicts the mechanism of release [43]. Based on the Korsmeyer–Peppas model, when *n* is ≤0.45 the EO release is controlled by diffusion (Fickian diffusion), for 0.45 < *n* < 0.89 the release mechanism is a combination of diffusion and swelling (non Fickian/anomalous diffusion). For *n* = 0.89 the mechanism is a zero-order release due to the swelling and the polymer matrix relaxation and, finally, when *n* > 0.89 there is an accelerated EO release (super case II transport) [44,45,46].

Using the logarithmic equation, the Korsmeyer–Peppas model is described as follows (Equation (6)):

(6) $$\mathrm{lg}\left(\frac{I_{t}}{I_{\infty }}\right)=lgK+n\;lgt$$

and allows to determine the EO release mechanism from the slope (*n*) of the graph lg(*I_t_*/*I_∞_*), vs. lg*t* (see Section 3.6), where lg*K* is the y-intercept and indicates *K*, the release rate constant.

### 2.13. Bacterial Strains

Microbiological analyses were performed on bacterial strains from established collections (i.e., *Enterococcus faecalis* ATCC 29212, *Enterococcus faecium* 64/3, *Escherichia coli* ATCC 25922, *Staphylococcus aureus* ATCC 43300) and on isolates recovered from clinical specimens, with resistance to vancomycin (i.e., VREfm-01, VREfm-02, VREfm-03, VREfm-04, VREfm-05) and linezolid (i.e., LREfm-01 and LREfm-02).

### 2.14. Determination of Minimal Inhibitory Concentration (MIC)

MICs were determined by a broth microdilution method in microtiter plates. Bacterial strains were diluted to a final concentration of 5 × 10^5^ CFUs/mL and mixed with different dilutions of EOs (*v*/*v*) in Mueller Hinton Broth (MHB), starting from 4% to 0.5% with 0.5% decrements. Plates were incubated for 24 h at 36 ± 1 °C, and MICs were defined as the lowest concentration (*v*/*v*) that inhibited visible bacterial growth.

### 2.15. Growth Curves

*E. faecium* 64/3 was used in bacterial growth curve experiments. In brief, the bacterial strain was 1 × 10^8^ CFUs/mL in MHB containing 0.5% of EOs and incubated for 24 h at 37 °C. Microbial growth was detected by measuring OD_690_ every hour. The experiment was performed in triplicate.

### 2.16. Bacterial Adhesion on PLA and PLA_EOs Films

The adhesion of *E. faecium* 64/3 on PLA and PLA_EOs films was assessed by CFUs/mL counts. Briefly, films were cut into 1 × 1.5 cm pieces and incubated for 1 h and 30 min at 36 ± 1 °C in MHB containing 1 × 10^8^ CFUs/mL of *E. faecium* 64/3. After incubation, the broth was removed, and the films were washed with saline to remove non-adherent cells. Then, the films were inserted into tubes containing 1 mL of water and 1% Tween 20 and vortexed to detach cells. To count CFUs, serial dilutions were plated in Brain Heart Infusion agar plates and incubated for 24 h at 36 ± 2 °C.

### 2.17. Statistical Analysis

The data of transport properties were statistically analyzed using SPSS Statistics 23. One-way analysis of variance (ANOVA) and least significant difference (LSD) procedures were applied for sample comparison. Differences were considered statistically significant at *p* < 0.05.

The data of the radical scavenging activity of EOs and PLA_EOs films were statistically analyzed using Microsoft Excel. One-way analysis of variance (ANOVA) and least significant difference (LSD) procedures were applied for sample comparison. Differences were considered statistically significant at *p* < 0.05.

## 3. Results and Discussion

### 3.1. Chemical Analysis of Hemp EOs

The yields of Fut and Carm hemp EOs, calculated on a dry weight basis (*w*/*w*), were 0.39% and 0.18%, respectively. The total chemical profile of the EOs, assessed through GC-MS analysis encompassed 39 identified compounds (see Table S1 and Figure S1). In both EOs, hydrogenated and oxygenated sesquiterpenes were more prevalent than monoterpenes. More precisely, the sesquiterpenes (*E*)-caryophyllene, α-humulene, and caryophyllene oxide were the most abundant compounds while, among monoterpenes, α-pinene and myrcene were present in noticeable amounts [26]. The exact composition of the EOs used in this study was found to be slightly different from those obtained from the same cultivars with different plant material status and extraction method in other studies [26]. Particularly, a higher abundance of sesquiterpenes can be related to the use of dried hemp biomass, since drying can cause a partial loss of the more volatile monoterpenes [47]. Regarding the extraction technique, our previous and preliminary investigation employing microwave-assisted extraction on the same inflorescences revealed that this alternative method yielded a lower amount of EOs and chemical compounds for both the varieties. For this reason, conventional hydrodistillation was selected and used to obtain the EOs for incorporation into the polymeric films formulated and characterized in this study. Moreover, differently from the current work, the EO from the hydrodistilled dry inflorescences of Carm was reported to be characterized by the predominance of monoterpenes and a much lower level of CBD [12], confirming that the cropping system, the environment, the harvesting time and storage conditions can influence the hemp EO chemical profile.

**Figure 2:**
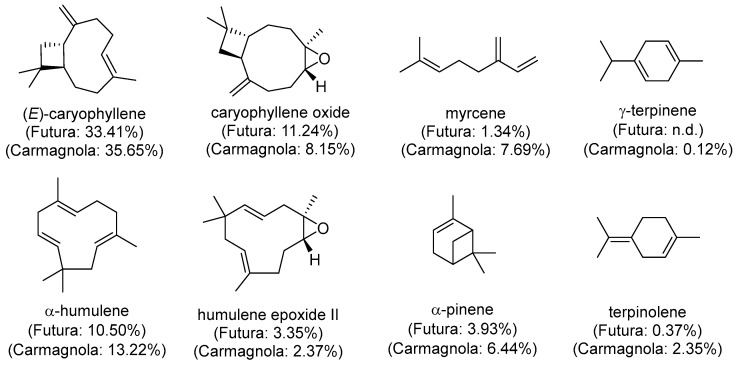
Components of EOs known for their antioxidant and antibacterial activity, with their relative peak area (n.d.: Not detectable).

Regarding the main components responsible for the antioxidant and antibacterial activities of hemp EOs, which are of interest for the corresponding sections, only a few of them have been specifically investigated so far. For the antioxidant properties, which should be in this case mainly related to the inhibition of hydrogen atom transfer (HAT) instead of single electron transfer (SET) mechanism [48], the most abundant component, that is (*E*)-caryophyllene (also called β-caryophyllene), showed a relevant activity (Figure 2) [49]. Myrcene and γ-terpinene also possess high radical scavenging potency [50,51], the former being much more present in Carm EO, while the latter is only a minor component of both EOs, when detectable (Figure 2).

Caryophyllene oxide, which is an abundant molecule especially in Fut EO, also demonstrated radical scavenging activity [52], and the same applies to α-humulene, whose content is high (particularly in Carm), as well as some of its derivatives [53]. Considering all of these data, Carm EO should intrinsically possess a higher antioxidant activity than Fut EO. However, it must be considered that other constituents feature allylic CH or CH_2_ groups, and very easily abstractable hydrogens adjacent to conjugated double bonds or between two C=C bonds (doubly allylic CH or CH_2_) (Figure S1). All these species can form stable radicals which are unable to propagate the radical chain reaction and may therefore act as good antioxidants [51].

As far as the antibacterial properties of terpenes are concerned, they are mainly due to the insertion and accumulation into phospholipid bilayers of bacterial membranes, altering their stability and leading to permeabilization or disruption, with a concomitant loss of cytoplasmic content [54,55]. Among monoterpenes, myrcene and α-pinene, both of which are much more abundant in Carm EO (Figure 2), showed antibacterial activity towards *listeria* spp. and enterococci [56]. On the other hand, the most abundant compound here (i.e., (*E*)-caryophyllene or β-caryophyllene) was proven to have relevant activity against various Gram-negative and positive bacteria [49,57,58], together with its derivative caryophyllene oxide [52], which is another abundant compound in both EOs.

### 3.2. ATR FTIR Analysis of EOs and Films

ATR FTIR spectroscopy was employed to investigate the interactions between PLA and the hemp derived essential oils Carm and Fut. As reported in the literature [59], distinguishing the characteristic bands of essential oils in PLA/EO films is challenging, as the oils tend to diffuse into the polymer matrix.

**Figure 3:**
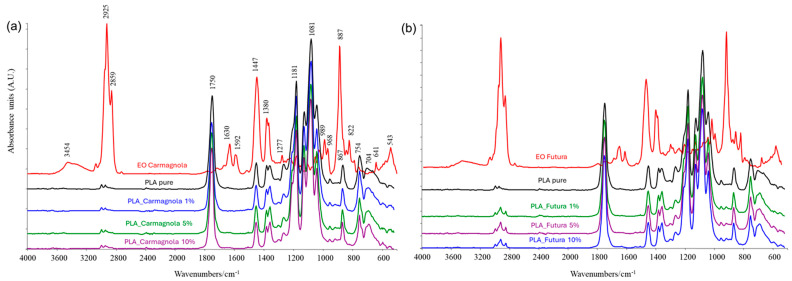
IR spectra of: (**a**) PLA_Carm-based systems compared to pure PLA and Carm spectra and (**b**) PLA_Fut-based systems compared to pure PLA and Fut EO spectra.

Figure 3 shows the representative spectra of the essential oils and neat PLA, compared with those of PLA_EO blend films containing 1, 5, and 10% wt EOs. Despite their intrinsically heterogeneous chemical composition, the hemp EOs exhibit highly comparable spectral features. A broad peak around 3450 cm^−1^ corresponds to O–H stretching vibrations of hydroxyl groups, confirming the presence of phenolic and alcoholic compounds. The region between 1100 and 1200 cm^−1^, attributed to C–O stretching and O–H in plane bending, together with the band around 1380 cm^−1^, further indicates the abundance of hydroxyl functionalities. The absorption band at approximately 2925 cm^−1^ arises from C–H stretching of aliphatic –CH_2_ and –CH_3_ groups, characteristic of alkyl chains within terpenoids. Meanwhile, the bands at 1630 and 1592 cm^−1^ can be assigned to aromatic C=C stretching vibrations, indicative of conjugated aromatic systems such as phenolic diterpenoids and flavonoids. The band at 887 cm^−1^ corresponds to the out of plane deformation of =C–H bonds in terpenes double bonds. Overall, the FTIR data confirm that Carm and Fut EOs are predominantly composed of bioactive terpenoids and phenolic derivatives known for their thermal and oxidative stability.

In the spectra of the PLA_EO films, the characteristic PLA bands (1750, 1447, 1380, 1181, 1081, 867, and 754 cm^−1^) are present, along with additional peaks associated with the essential oils. In PLA_Carm films, increasing the EO content leads to a progressive intensification of the peaks at 2859 cm^−1^ (very weak, appearing more as a shoulder than a distinct peak) and 887 cm^−1^, shifted to 898 cm^−1^, reflecting the contribution of the oil. In PLA_Fut films, however, the presence of the EO is more pronounced and largely independent of concentration: even at 1% wt EO, the peaks at 2922, 2859, and 898 cm^−1^ are clearly visible.

The infrared spectra of the PLA_EOs samples, including the magnified spectral ranges, are provided in the Supplementary Materials (Figures S2 and S3).

### 3.3. TGA of EOs and Films

**Figure 4:**
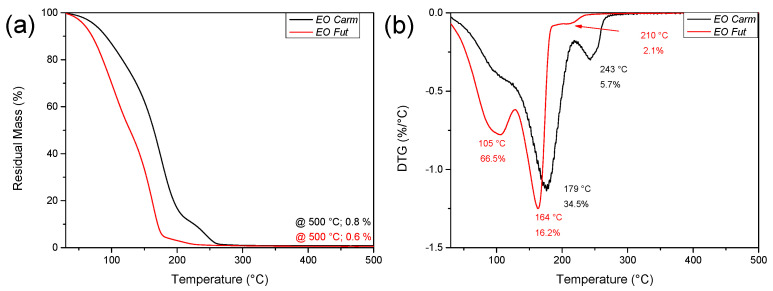
Residual mass (**a**) and derivative curves (**b**) of the EOs weight loss.

The thermal stability of EOs under an inert atmosphere was analyzed by thermogravimetric analysis (TGA); Figure 4 shows the weight loss as a function of temperature as well as the curve of its derivative for the two EOs. The EOs are characterized by a multistep degradation; being composed of volatile constituents, they showed in fact a limited thermal stability, like other plant metabolites previously studied, such as oleuropein [60]. The initial mass loss up to 105 °C is attributed to water and volatile substance evaporation. The EO from Carm variety displayed a higher thermal stability than that from Fut, being the maximum peak of degradation centred at 179 °C for EO Carm and at 164 °C for EO Fut. The thermal profiles show a shoulder at 210 °C and at 245 °C for Fut and Carm, respectively. The increased thermal stability of EO Carm compared to EO Fut can be related to the more complex chemical composition, as observed in previous studies.

**Figure 5:**
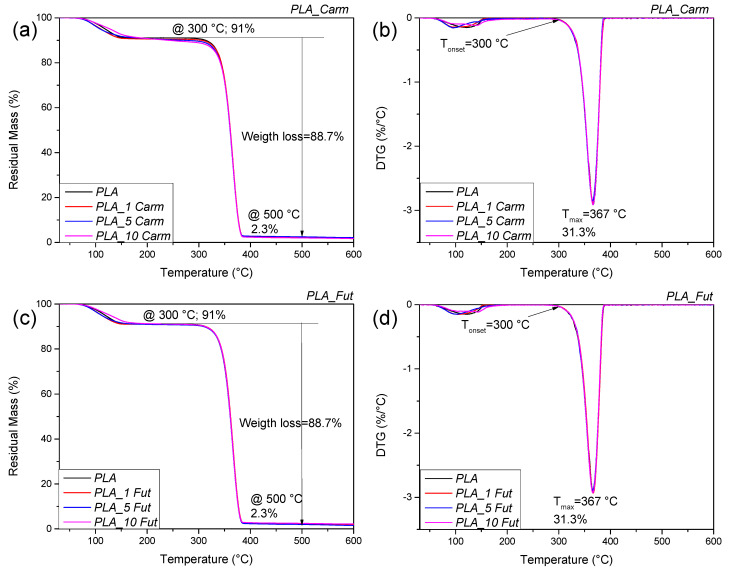
Residual mass (**a**,**c**) and derivative curves (**b**,**d**) of PLA_Carm and PLA_Fut-based films.

PLA_EOs films TGA analysis was performed to evaluate the effect of the different concentrations of both EOs on the thermal stability of the biobased matrix. The graphs related to the weight loss as a function of temperature and the curves of its derivative (DTG) for the two PLA_EO films are reported in Figure 5. PLA presented a multistep degradation process: the first degradation peak was centred at 113 °C, likely due to the solvent evaporation and humidity residual related to the storage conditions; the second peak presented an initial degradation temperature (T_onset_) of 300 °C, while the maximum degradation temperature (T_max_) was centred at 367 °C, indicating the PLA matrix degradation [34]. In conclusion, the addition of EOs, regardless of its concentration, seems not to produce any detectable modification in the thermal profile.

### 3.4. DSC of Films

**Figure 6:**
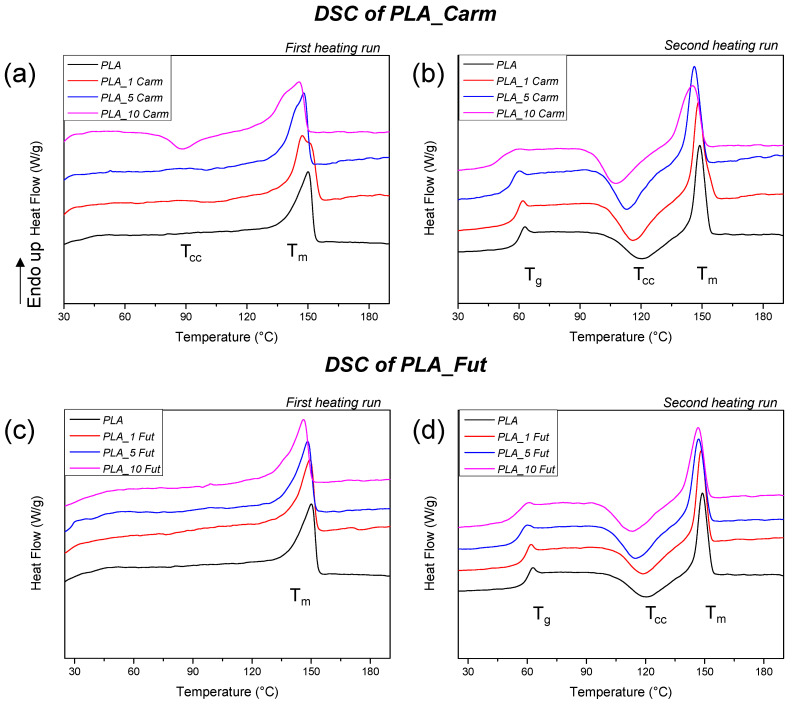
Thermograms of first (**a**,**c**) and second heating (**b**,**d**) of PLA_Carm and PLA_Fut formulations.

DSC analysis was conducted in order to determine the EOs effect (typology and content) on the T_g_, T_cc_ and T_m_ phenomena of PLA-based formulations. The values of T_g_, T_cc_, T_m_, as well as ΔH_cc_ and ΔH_m_ for PLA and PLA_EOs films are summarized in Table 1, while Figure 6 shows the thermograms obtained from the first and second heating scans of PLA. The DSC curve of neat PLA during the first heating scan displayed the endothermic melting peak centred at T_m_ (150 °C) (Figure 6a,c) [61]. A shoulder can be observed around the melting peak for PLA_Carm films; this pattern could be attributed to the formation of two different crystal structures as reported in the literature [62,63]. Table 1 reports only the T_m_ data corresponding to the maximum in the curve.

The incorporation of EOs into the polymer matrix caused a shift in the glass transition temperature (T_g_) towards lower values at the second heating scan (Table 1 and Figure 6b,d) as compared to pure PLA film, which is not visible in the first heating scan (Figure 6a,c). A similar trend was observed for the melting temperature (T_m_): Both the first and second heating scans showed a reduction in T_m_ upon the addition of EOs, in agreement with previous research [64]. Among the formulations tested, the PLA_10 Carm sample exhibited the most pronounced decrease in T_m_ relative to neat PLA. In the second heating scan, the T_cc_ of neat PLA is centred around 120 °C and, in the case of PLA_EOs formulations, the value is shifted to lower temperature because of EOs addition [65]. According to the literature, all the considered temperature shifts toward lower values are the result of plasticising effects determined by EOs added in the PLA matrix [12].

### 3.5. Antioxidant Activity of EOs and PLA_EOs Films Assessed via DPPH Assay

**Figure 7:**
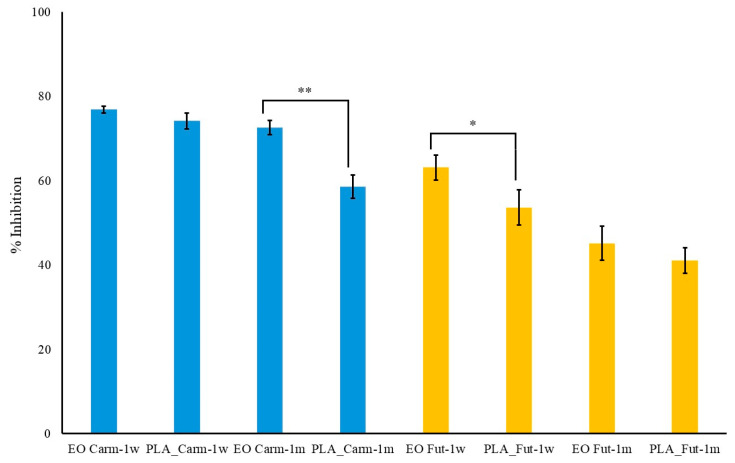
Radical scavenging activity of EOs and PLA_EOs films (blue: Carm; yellow: Fut) at the same equivalent concentration (1.56 mg mL^−1^) after one week (1 w) and one month (1 m). Data are mean ± standard deviation and were analyzed by one-way ANOVA: *N* = 3, * *p* < 0.05, ** *p* < 0.01.

The present study employed the DPPH (2,2-diphenyl-1-picrylhydrazyl) assay to evaluate and compare the radical scavenging activities of EOs and PLA_EOs films at a standardized concentration of 1.56 mg mL^−1^. The results, depicted in Figure 7, illustrate the percentage inhibition of DPPH radicals for both free EOs and PLA_EOs films. The findings demonstrate that the antioxidant activity of the EOs is effectively retained within the PLA_EOs films even after a period of one week. Notably, the data indicate that the combination of polymer with EOs does not significantly reduce the radical scavenging capacity of the EOs for the PLA_Carm, while a slight reduction of about 10–15% was observed for the EO Fut in PLA films. However, considering the low decrease in DPPH inhibition, the results obtained suggested that PLA acts as a suitable carrier that preserves antioxidant functionality over time, as observed for PLA/PCL films loaded with thymol and/or carvacrol [66].

Furthermore, comparative analysis between the two types of EOs Carm and Fut reveals that Carm exhibits significantly superior antioxidant activity in both its free EO form (after one week, *p* < 0.01; after one month, *p* < 0.001) and when incorporated into PLA films (after one week and one month, *p* < 0.01; (Figure 7). This enhanced activity may be attributed to the inherent chemical composition of Carm EO, which is characterized by larger amounts of known active compounds in comparison to Fut EO (i.e., (*E*)-caryophyllene, myrcene and γ-terpinene) (Figure 2).

For long-term tests, films and essential oils were stored at room temperature in the dark for one month. After such a period, the decrease in activity of EO Carm within the PLA_EO film was statistically significant (*p* < 0.01) with respect to the same EO in free form, whereas Fut in PLA_EO film showed no statistically significant decrease (*p* > 0.05) (Figure 7).

Interestingly, there is an approximately 18% decrease in the antioxidant activity of Carm within the PLA film compared to that of the EO in free form. Conversely, no significant differences are observed for Fut. Moreover, it is worth noting that for both films a final decrease of 20%, only at different times (one month for Carm and one week for Fut) has been found.

The present results obtained with the DPPH assay favorably compare with those obtained with other PLA films. In fact, when PLA films were coated with chitosan or chitosan/sodium caseinate filled with rosemary essential oil up to a concentration of 2% *w*/*v*, the best antioxidant activity was of 6% [67]. In addition, when oregano EO was added to PLA films during extrusion, the activity at the highest percentages employed (5% and 10% EOs) was only slightly worse than that of Carm EO, but the corresponding films also evidenced an excessive plasticising effect [68]. Better results for PLA-based films could be obtained only when tea tree oil was added to films made of poly(lactic acid), zein, and polyethylene glycol (PEG) [69].

In conclusion, the DPPH assay confirms that PLA_Carm and PLA_Fut films maintain good antioxidant properties of EOs over time, with Carm EO showing markedly higher radical scavenging efficacy than Fut EO. These results underscore the potential of PLA_EO films as stable, effective antioxidant materials for applications in packaging or biomedical fields, where sustained free radical scavenging activity is required. However, even if the DPPH test provides this first positive indication, future studies employing more comprehensive test models for evaluating radical scavenging activity when the films are in contact with the food matrix [67,68,69] will be carried out.

### 3.6. Kinetic Release of Oils from PLA

**Figure 8:**
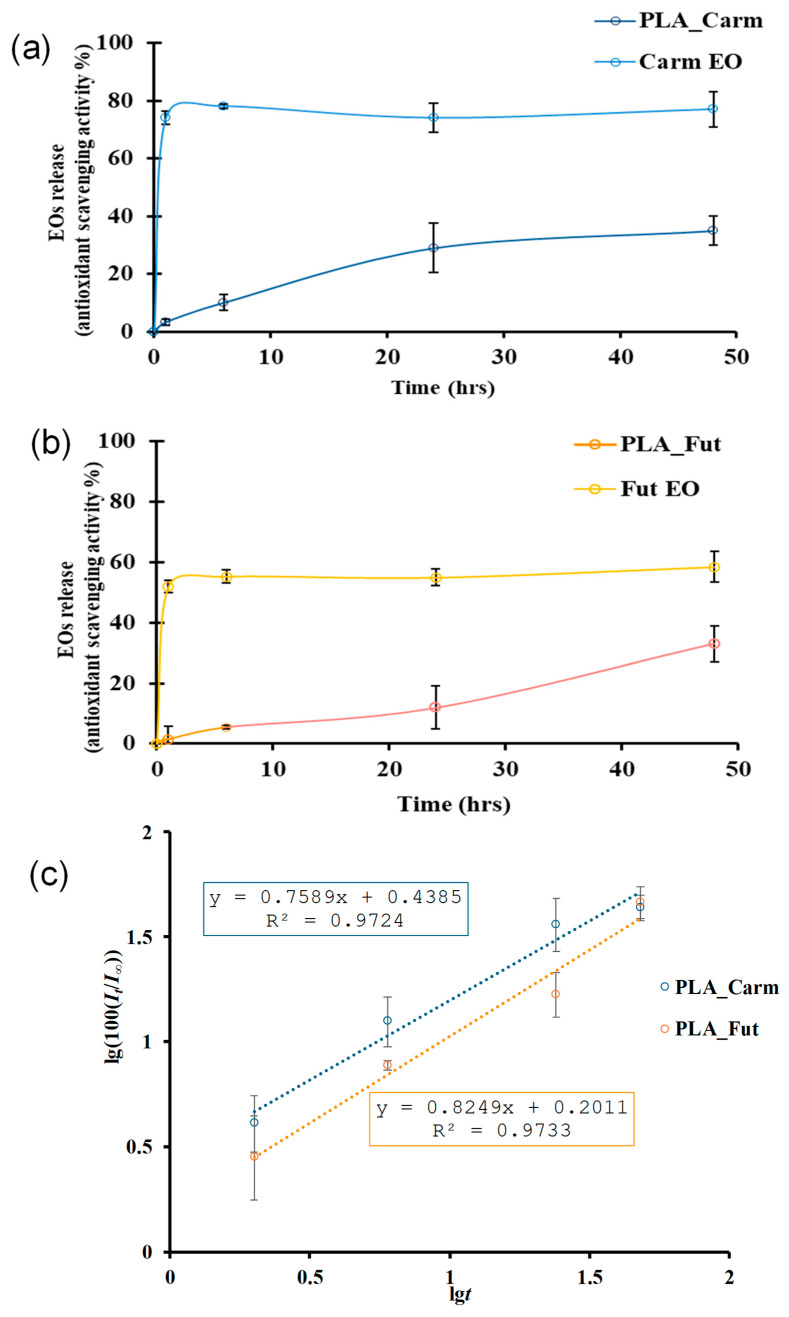
EOs released from PLA matrix and in free form for PLA_Carm (**a**) and PLA_Fut (**b**); lg(100(*I_t_*/*I_∞_*)) as a function of lg*t* for PLA_Carm and PLA_Fut (**c**). Data are mean ± standard deviation.

The kinetic release study demonstrated that the migration of EOs from PLA films is strongly influenced by the polarity of the receiving medium. Release was monitored using the DPPH radical scavenging assay as an indirect quantification method, to determine the amount of antioxidant compounds released over time. In pure water, no detectable release was observed during the monitored time interval. This finding indicates a very limited transfer of EOs constituents from the hydrophobic PLA matrix into a highly polar aqueous phase, likely due to unfavorable oil–water partitioning and low EOs solubility. In contrast, when a less polar simulant (water/ethanol 1:4 *v*/*v*) was used, EOs’ release was significantly enhanced. This behavior can be attributed to improved EOs solubility in the hydroalcoholic medium and possible partial swelling of the PLA matrix, which may facilitate molecular diffusion. As shown in Figure 8a, for Carm EO, the amount released after 1 h resulted in approximately 74% DPPH inhibition, corresponding to a concentration of 1.56 mg mL^−1^ (Figure S4A). This value matches the expected theoretical concentration if the total EO content were to be fully released into the medium. This suggests complete release within the first hour, which is consistent with the burst phenomenon. Fut EO displayed similar behaviors: a complete release of the EO yielded approximately 55% DPPH inhibition at the same theoretical concentration of 1.56 mg mL^−1^, reflecting its lower intrinsic radical scavenging activity compared to Carm at equivalent concentrations (Figure S4B). Nevertheless, a burst effect was also observed for Fut EO in its free form (Figure 8b).

In Figure 8c, in order to avoid negative values, lg(100(*I_t_*/*I_∞_*)) is reported instead of lg(*I_t_*/*I_∞_*). As expected on the basis of the similar composition of Carm and Fut EOs, the release exponents values for both EOs are very close and are 0.45 < *n* < 0.89 (from the equations in Figure 8c: *n* = 0.76 for PLA_Carm and *n* = 0.82 for PLA_Fut) and then, for both EOs, the release mechanism from the PLA matrix is a combination of diffusion and swelling.

When incorporated into PLA films, both EOs exhibited a slower and more controlled release profile, although migration remained more pronounced during the initial hours. This early-stage release likely arises from surface-associated or weakly entrapped molecules, whereas the subsequent release phase appears to be governed by diffusion through the polymer bulk. The differences observed between Carm and Fut EOs can be attributed to their distinct chemical compositions and/or specific interactions with the PLA matrix, which influence both diffusion kinetics and antioxidant scavenging activity. Overall, PLA behaves as a reservoir system that mitigates the burst phenomenon observed for free EOs and enables sustained release over time. Similar release behavior has been reported in other systems based on PLA films loaded with other essential oils [40,70].

### 3.7. Microscopy Examinations

**Figure 9:**
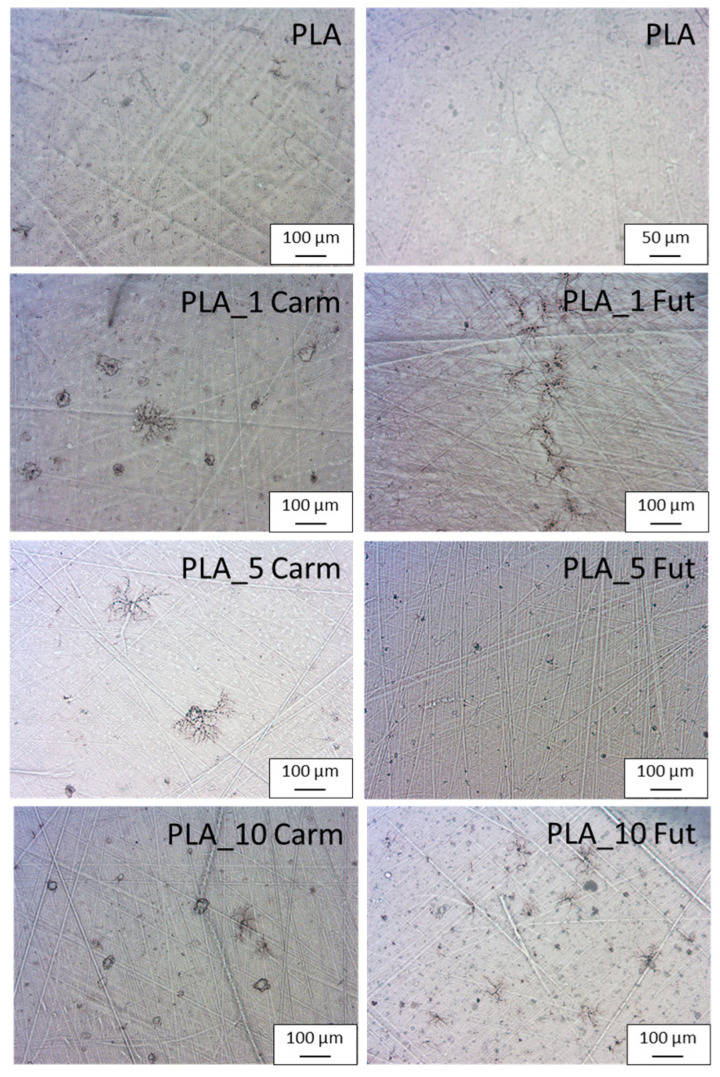
Optical microscopy images of PLA_EOs-based formulations.

The microstructure of the external surfaces of pure PLA and PLA_EOs films represented in Figure 9 resulted to be smooth and homogeneous [71]. The presence of both EOs did not alter the microstructure of the polymeric films, evidencing a rather homogenous distribution of the EOs into the polymeric matrix. This distribution is of particular significance regarding the barrier properties of polymer films. In fact, the absence of phase separation or micro-domains prevents the formation of preferential diffusion pathways that could facilitate the permeation of gases or water vapour [72]. The structural integrity of the polymeric films allows, in some cases, slightly enhanced barrier properties [34].

**Figure 10:**
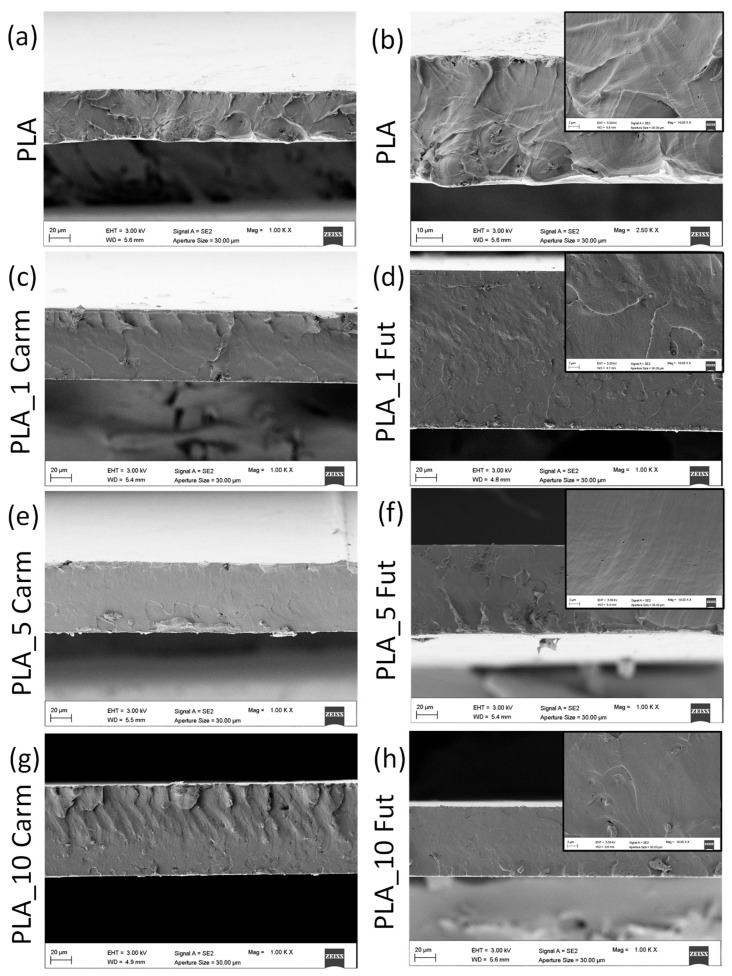
FESEM investigation of fractured surfaces of PLA and PLA_EOs based formulations. PLA (**a**,**b**); PLA_1 Carm (**c**); PLA_1 Fut (**d**); PLA_5 Carm (**e**); PLA_5 Fut (**f**); PLA_10 Carm (**g**) and PLA_10 Fut (**h**).

Figure 10 shows the microstructure of the cryo-fractured cross-sectional surfaces of PLA films analyzed by FESEM to evaluate the effect of EO additions at different concentrations. PLA film fractured surface appeared homogeneous and uniform, as it is typical for PLA films based on semi-crystalline virgin PLA [73]. A similar microstructure was also revealed for PLA_EOs-based films: the cross sections appear, in fact, homogeneous, uniform, smoother with a slight presence of non-interconnected porosity, and with fewer irregularities [74]. Phase separations are not detected (Figure 10b–h, see also the insert), showing good processability of PLA_EOs films.

### 3.8. Mechanical Properties

**Figure 11:**
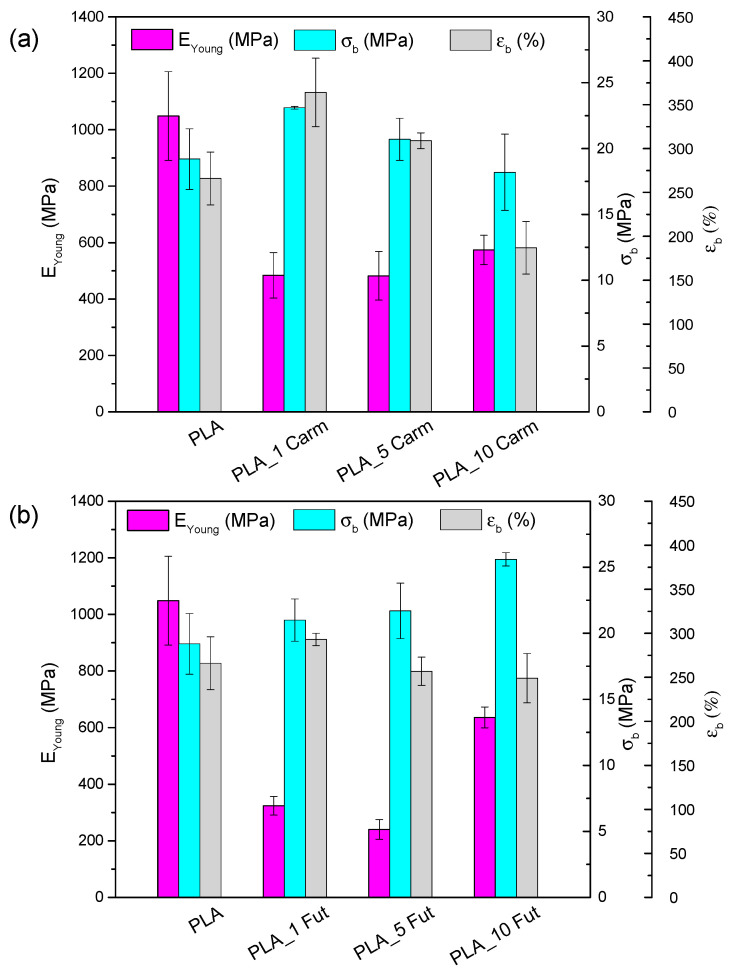
Mechanical characteristic parameters obtained by the tensile test for PLA, PLA_Carm (**a**) and PLA_Fut (**b**) based films.

Figure 11 summarizes the results of mechanical characterization obtained through tensile tests. E_Young_, σ_b_ and ε_b_ values for PLA film are comparable with those reported in the literature [75,76]. PLA_Carm (Figure 11a) and PLA_Fut (Figure 11b)-based films exhibit a reduction in E_Young_, an improvement in stretchability, evidenced by an increase in εb with respect to the neat PLA film. This behavior is attributed to the presence of essential oils, which act as plasticizers in the polymeric matrix, as previously observed in studies combining PLA with other essential oils [11,77]. However, the impact of EO addition is concentration-dependent; specifically, the incorporation of both EOs at 10% wt induces an increase in rigidity and stress at break.

### 3.9. Transport Properties

Pervaporation measurements were carried out for PLA and PLA_EOs films. These measurements allow for determining the influence of the EO presence on the transport properties such as the permeability and diffusivity of water through the film. The permeability coefficient (P) is the product of gas flow (J) and film thickness (L) divided by the activity (or pressure) difference between the two faces of the film. The diffusion coefficient (D) is evaluated from the permeation curve with normalized flow (J/J_st_) as a function of time, where J_st_ represents the limit flow at the steady state. On the Fick’s law basis, one can calculate the time-lag diffusion coefficient (D_L_) from the transient permeation curve at J/J_st_ = 0.6167 for which t = tL by the equation [78]:

(7) $$D_{L}=\frac{L^{2}}{6t_{L}}$$

and the diffusion at the inflexion point I (D_I_) at J/J_st_ = 0.24 for which t = t_0.24_ by the equation:

(8) $$D_{I}=\frac{0.091\cdot L^{2}}{t_{0.24}}$$

The values of P, D_I_, and D_L_ were calculated for pure PLA and PLA_EOs films (Table 2).

The water permeability of the polymer film depends on the solubility parameter linked to the affinity between the polymer and permeant, and on the water diffusivity, mainly related to the structure and particularly to the density connected to the degree of crystallinity and to the film stiffness (glassy or rubbery state). At first heating scan, no variations in crystallinity degree were registered adding different content and typology of EOs in PLA-based films except for PLA_10 Carm (as summarized in Table 2). The presence of EOs in PLA film contributes to a decrease in the water permeability coefficient. When PLA contains 1% EOs, only a very small barrier effect is observed for the Fut (5% of barrier improvement factor (BIF) for PLA_1 Fut), while it is absent for Carm (PLA_1 Carm). In case of EO Carm containing film, the highest BIF is obtained for PLA_10 Carm, while for EO Fut containing film the highest BIF value is obtained for PLA_5 Fut (15% BIF). However, this enhancement does not appear to be concentration dependent, as the BIF values reported are comparable (Table 2).

The comparison of *D_L_* and *D_I_* coefficients (Table 2) shows that *D_L_* value is higher than *D_I_* value for all PLA films. As the former value corresponds to a latter period of the transient state (*J/J_st_* = 0.62), the smaller *D_I_* value means that the water diffusion increases during the permeation process. Generally, such diffusion increase is attributed to the plasticization of the material by water which leads to an increase in the material free volume. All permeation curves were fitted to the well-known exponential law of diffusion [78]:

(9) $$D=D_{0}e^{\gamma C}$$

where *D*_0_ is the limit diffusion coefficient, *γ* is the plasticization coefficient and *C* is the local permeant concentration.

**Figure 12:**
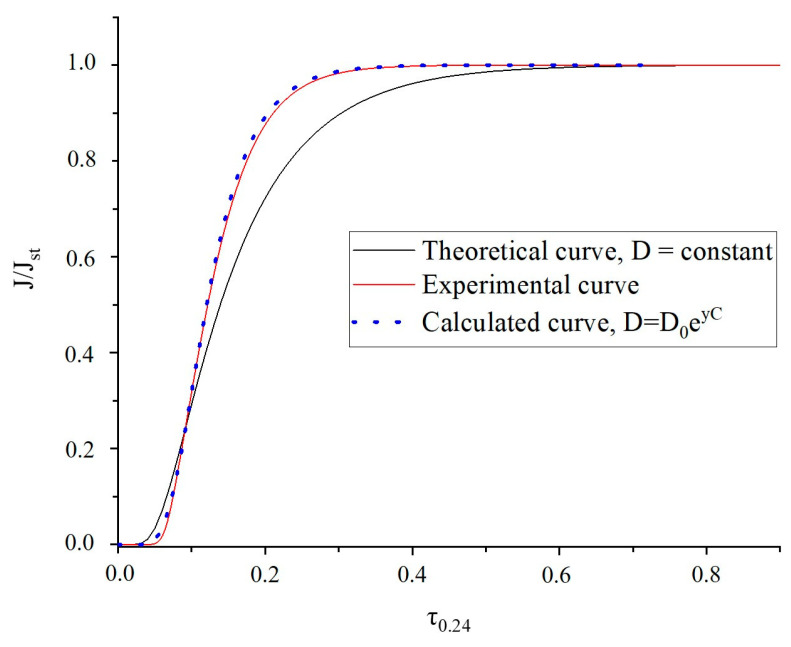
Experimental and theoretical permeation flux curves of the PLA film in the dimensionless scale of flux and time.

From the dimensionless scale of flux curve *J*/*J_st_* versus time τ_0.24_ depicted in Figure 12, an excellent agreement is obtained between experimental and calculated flux curves by using the concentration-dependent diffusion coefficient (Equation (9)). This result indicates that the diffusion coefficient is not constant but increases with penetrant concentration, suggesting a plasticization effect and a concentration-dependent transport behavior. From the curves obtained it was possible to determine the mean integral diffusion coefficient *<D>* and the plasticization factor *γC_eq_*, with *C_eq_* as the limit condition of concentration. One can see that the plasticization factor (*γC_eq_*) remains practically constant except for PLA_5 Carm film (Table 2). The obtained *<D>* values demonstrate that the reduction in the water permeability for the EOs-containing films can be attributed to the decrease in the water diffusivity.

Therefore, it can be deduced that the permeability decrease can be explained by the reduction in the affinity between the water molecules and the resulting material, due to a decreased solubility coefficient by the presence of hydrophobic EOs.

### 3.10. Antimicrobial Assays

MICs values of the different EOs were reported in Table 3. Among the reference strains, both EOs showed the lowest MICs values against *E. faecium* and high MICs values for *E. coli*. These data were in accordance with previous works which showed good antimicrobial activity of EOs against Gram-positive species while the compounds were less effective against Gram-negatives, probably due to the presence of the outer membranes in these last species [27,28].

Given the higher activity against *E. faecium*, EOs activity was tested against some isolates resistant to vancomycin and linezolid, two antibiotics commonly used in microbial infections sustained by enterococci. As already reported, Carm was more active compared to Fut [79]. The detected MICs values differed among the clinical strains, and the effectiveness of the EOs was strain-dependent, suggesting that the compounds activity was not affected by antibiotic resistance mechanisms.

**Figure 13:**
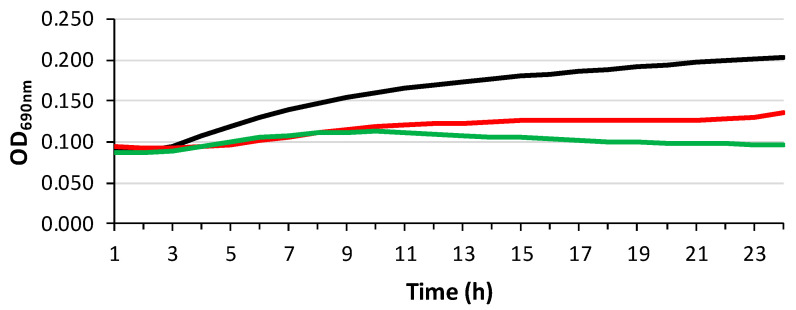
Time kill curves of EOs against *E. faecium* 64/3. Black curve, positive control; red curve Fe, 0.5% (*w*/*w*) Fut EO; green curve, 0.5% (*w*/*w*) Carm EO.

To better evaluate the effectiveness of EOs, a growth curve assay was performed against *E. faecium* 64/3 using 0.5% (*w*/*w*) of both Fut and Carm EOs (Figure 13). Both EOs hampered the microbial growth, determining a reduction in viable cells after 24 h compared to control. The EOs showed a bacteriostatic activity that could also be combined with antibiotic activity. Indeed, it has been demonstrated that natural compounds and essential oils can be successfully used in combination with antibiotics to obtain a synergic activity [80].

Further experiments should be performed to detect if higher concentrations of EOs could have a bactericidal activity and if combinations with antibiotics have synergic effects.

The adhesion experiments on PLA_EO films did not reveal differences in CFU counts when comparing the different formulations and the PLA itself. The adhesion process of bacteria on surfaces is a multifactorial process and includes three different phases, where the second one is a reversible adhesion due to Lifshitz-van der Waals interactions and electrostatic interactions [81]. The incorporation of EOs in PLA films, although reducing the electrostatic charge as demonstrated by the reduction in water permeability, did not prevent the binding of the bacteria to the material. This is consistent with previous studies on other EOs or extracts in PLA films, which showed no inhibition for rice bran extract [82], while films with 10% oregano EO were active only against *Salmonella enterica* but inactive towards many other strains [68]. However, relevant antimicrobial activities were found for polylactic acid-polyhydroxybutyrate films when the most powerful antibacterial compound in fennel oil was by far the main component [83], or when pure active compounds were added to PLA-based bilayer films [84]. In addition, when PLA films also contained nanostructured cellulose together with EOs, the growth of the adherent bacteria was prevented, avoiding contamination spread, preserving the food, and increasing the shelf-life of the packed material [85,86]. Therefore, further experiments should be performed after slight modifications of the present formulation in order to increase the release of EOs components, especially when in contact with a hydrophilic environment, and then improve the antibacterial efficacy, before evaluating the performances of PLA_EOs films in preserving real foods [87].

## 4. Conclusions

In this study, PLA-based films combined with hemp Eos from Carm and Fut cultivars at different concentrations (1, 5 and 10% wt) were successfully developed through solvent casting and characterized in terms of chemical, morphological, and thermal properties, as well as antioxidant activity and transport properties. The results demonstrated that the addition of both the Eos did not compromise the morphological aspect of the PLA films, while a variation on glass and melting temperature was recorded due to the plasticising effect of EOs, also confirmed by tensile tests. In fact, the addition of essential oils induced an improvement of deformation at break and a reduction in stress at break and Young’s modulus for PLA_EOs films with respect to neat PLA. The presence of essential oils in PLA film contributed to a decrease in the water permeability coefficient, especially as the concentration of EOs increases. In addition, antimicrobial tests revealed that hemp essential oils have notable antimicrobial properties, particularly against Gram-positive bacteria, although the activity against Gram-negative bacteria was limited. However, the lack of antibacterial activity of PLA_EOs films highlighted the necessity for some structural modifications aimed at improving release kinetics, which are currently underway in our laboratory.

The radical-scavenging activity of Carm and Fut Eos was preserved after incorporation in PLA-based films. Although the results indicate that both the Eos represent promising, sustainable ingredients for developing active PLA packaging materials with improved antioxidant properties, Carm exhibits higher activity than Fut.

## Figures and Tables

**Table 1:** Thermal properties for PLA and PLA_EOs films obtained by DSC analysis.

**Formulations**	**First heating run**					
	T_g_ (°C)	ΔH_cc_ (J g^−1^)	T_cc_ (°C)	ΔH_m_ (J g^−1^)	T_m_ (°C)	X_m_ (%)
***PLA***	-	-	-	24.4 ± 0.3	150.4 ± 0.1	26.2 ± 0.3
***PLA_1 Carm***	-	1.1 ± 0.1	102.8 ± 0.5	25.3 ± 0.4	147.1 ± 0.2	26.3 ± 0.4
***PLA_5 Carm***	-	1.6 ± 0.2	101.0 ± 0.3	22.3 ± 0.3	147.8 ± 0.4	23.4 ± 0.4
***PLA_10 Carm***	-	9.2 ± 0.1	88.3 ± 0.1	20.8 ± 0.6	145.3 ± 0.6	13.8 ± 0.6
***PLA_1 Fut***	-	-	-	22.9 ± 0.4	149.4 ± 0.1	24.9 ± 0.5
***PLA_5 Fut***	-	-	-	20.9 ± 0.7	148.1 ± 0.1	23.7 ± 0.8
***PLA_10 Fut***	-	-	-	21.9 ± 0.2	146.4 ± 0.2	26.1 ± 0.3
**Formulations**	**Second heating run**					
	T_g_ (°C)	ΔH_cc_ (J g^−1^)	T_cc_ (°C)	ΔH_m_ (J g^−1^)	T_m_ (°C)	X_m_ (%)
***PLA***	60.3 ± 0.2	15.0 ± 0.9	120.1 ± 0.1	16.1 ± 0.7	148.7 ± 0.1	1.2 ± 0.2
***PLA_1 Carm***	59.4 ± 0.2	18.1 ± 0.1	116.1 ± 0.2	19.7 ± 0.2	147.9 ± 0.5	1.1 ± 0.2
***PLA_5 Carm***	56.2 ± 0.3	19.2 ± 0.2	112.8 ± 0.4	20.1 ± 0.1	145.9 ± 0.3	1.1 ± 0.2
***PLA_10 Carm***	52.0 ± 0.5	18.1 ± 0.7	107.4 ± 0.6	18.9 ± 0.7	144.8 ± 0.4	1.1 ± 0.1
***PLA_1 Fut***	59.4 ± 0.2	17.6 ± 0.5	119.2 ± 0.3	18.9 ± 0.4	148.1 ± 0.1	1.1 ± 0.2
***PLA_5 Fut***	56.4 ± 0.2	18.1 ± 0.6	114.6 ± 0.4	18.9 ± 0.7	146.9 ± 0.1	1.0 ± 0.1
***PLA_10 Fut***	55.1 ± 0.1	18.1 ± 0.4	112.4 ± 0.4	18.8 ± 0.4	146.5 ± 0.3	1.1 ± 0.1

**Table 2:** Water transport properties through PLA and PLA_EOs films (*p* < 0.05).

	*P* (Barrer *)	*D*_0_*·*10^10^ (cm^2^·s^−1^)	*D_I_·*10^10^ (cm^2^·s^−1^)	*D_L_·*10^10^ (cm^2^·s^−1^)	*<D>·*10^10^ (cm^2^·s^−1^)	*γC_eq_*	*γ* (cm^3^·mmol^−1^)	*C_eq_* (mmol·cm^−3^)	*BIF* (%)
**PLA**	3027 ± 300	1.35 ± 0.72	2.87 ± 0.14	3.83 ± 0.39	6.79 ± 1.48	2.74 ± 1.11	6.8 ± 4.2	0.48 ± 0.14	-
**PLA_1 Carm**	3056 ± 412	0.57 ± 0.04	2.60 ± 0.18	4.02 ± 0.25	8.02 ± 0.52	4.06 ± 0.01	10.04 ± 1.89	0.41 ± 0.08	−1
**PLA_5 Carm**	2543 ± 132	1.17 ± 0.11	2.25 ± 0.01	2.87 ± 0.14	4.95 ± 0.28	2.42 ± 0.21	4.58 ± 0.44	0.53 ± 0.01	16
**PLA_10 Carm**	2499 ± 44	1.23 ± 0.11	1.78 ± 0.35	2.47 ± 0.06	4.55 ± 0.98	1.86 ± 0.04	2.81 ± 0.03	0.66 ± 0.02	17
**PLA_1 Fut**	2889 ± 180	1.62 ± 0.29	3.31 ± 0.28	4.32 ± 0.35	7.62 ± 0.23	2.59 ± 0.22	6.58 ± 0.01	0.39 ± 0.03	5
**PLA_5 Fut**	2577 ± 225	1.63 ± 0.09	2.41 ± 0.34	2.82 ± 0.46	4.40 ± 0.94	1.72 ± 0.24	2.85 ± 0.72	0.61 ± 0.07	15
**PLA_10 Fut**	2623 ± 130	1.68 ± 0.15	2.51 ± 0.16	2.94 ± 0.17	4.61 ± 0.21	1.78 ± 0.06	3.02 ± 0.16	0.59 ± 0.05	13

* 1 Barrer = 10^−10^ cm^3^ (STP)·cm·cm^−2^·s^−1^·cmHg^−1.^

**Table 3:** MIC values of the EOs against selected bacterial strains.

Isolate	MICs (% *v*/*v*)	
	Futura EO	Carmagnola EO
*E. coli* ATCC25922	>4%	>4%
*E. faecium* 64/3	≤0.5%	≤0.5%
*E. faecalis* ATCC29212	2%	0.5%
*S. aureus* ATCC43300	>4%	3%
VREf-01	≤0.5%	≤0.5%
VREf-02	2%	2%
VREf-03	1%	≤0.5%
VREf-04	>4%	2%
VREf-05	≤0.5%	≤0.5%
LREf-01	1%	1%
LREf-02	2%	≤0.5%

## Data Availability

The original contributions presented in this study are included in the article/Supplementary Material. Further inquiries can be directed to the corresponding author(s).

## Supplementary Materials

The following supporting information can be downloaded at: https://www.mdpi.com/article/10.3390/polym18070824/s1, Table S1: Chemical composition of Futura 75 and Carmagnola CS hemp EOs. Figure S1: Chemical structures of all the 39 compounds identified in Futura 75 and Carmagnola CS hemp EOs; Figure S2: Selected zones of IR spectra of PLA_Carmagnola CS-based systems compared to pure PLA and Carmagnola EO spectra (see main text for the discussion). Figure S3: Selected zones of IR spectra of PLA_Futura 75-based systems compared to pure PLA and Futura 75 spectra (see main text for the discussion). Figure S4: Calibration curves relative to DPPH inhibition of different concentrations of EOs (**A**, Carmagnola CS; **B**, Futura 75) in water/ethanol 1:4, *v*/*v*.
