# Supplementary material for: Hemp Essential Oils as Novel Antioxidant and Bacteriostatic Agents in PLA-Based Packaging
Source: Polymers (Basel). 2026 Mar 27;18(7):824. doi: 10.3390/polym18070824 (PMC13074719; doi:10.3390/polym18070824)
Supplement: Supplementary file 1 [file polymers-18-00824-s001.zip › polymers-4102862-supplementary.pdf]

Supplementary Materials

Hemp Essential Oils as novel antioxidant and bacteriostatic agents in PLA-based packaging

Eugenia Mazzara[1a], Annafelicia Civitavecchia[2a], Pierluigi Stipa[2], Cristina Minnelli[3], Emiliano Laudadio[2], Tiziano Bellezze[2], Pietro Forcellese[4], Samuele Rinaldi[3], Kateryna Fatyeyeva[5], Gianluca Morroni[6,7], Gloria D'Achille[6], Simona Sabbatini*[2b], Francesca Luzi*[2b]

[1] Food Chemistry and Technology Department, Teagasc Food Research Centre, Ashtown, D15 DY05, Dublin 15, Ireland; Eugenia.Mazzara@teagasc.ie

[2] Department of Science and Engineering of Matter, Environment and Urban Planning, Università Politecnica delle Marche, Via Brecce Bianche, 60131 Ancona, Italy; a.civitavecchia@staff.univpm.it; p.stipa@staff.univpm.it; e.laudadio@staff.univpm.it; t.bellezze@staff.univpm.it;s.sabbatini@staff.univpm.it; f.luzi@staff.univpm.it

[3] Department of Life and Environmental Sciences, Università Politecnica delle Marche, Via Brecce Bianche, 60131 Ancona, Italy; c.minnelli@staff.univpm.it; s.rinaldi@staff.univpm.it

[4] Department of Theoretical and Applied Sciences, eCampus University, Via Isimbardi, 10, 22060 Novedrate, Italy; pietro.forcellese@uniecampus.it

[5] Normandie Univ, UNIROUEN, INSA ROUEN, CNRS, Polymères Biopolymères Surfaces (PBS), 76000 Rouen, France; kateryna.fatyeyeva@univ-rouen.fr

[6] Departements of Biomedical Sciences and Public Health, Università Politecnica delle Marche, Via Tronto 10/A, 60126 Ancona, Italy; g.morroni@staff.univpm.it; g.dachille@staff.univpm.it

[7] Microbiology Laboratory, Azienda Ospedaliero Universitaria delle Marche, via Conca 71, 60126, Ancona

a E. M. and A. C. did equal contribution to the work and are co-first authors.

b S. S. and F. L. did equal contribution to the work as co-last authors.

* Correspondence: Simona Sabbatini, s.sabbatini@staff.univpm.it and Francesca Luzi, f.luzi@staff.univpm.it; Tel. +39 071 2204722; 60131 Ancona, Italy

TABLE OF CONTENTS

Chemical composition of Futura 75 and Carmagnola CS hemp EOs................................ 3

Additional figures for FT-IR spectra 6

Calibration curves for Carmagnola CS and Futura 75 in water/ethanol 1:4, v/v 8

Chemical composition of Futura 75 and Carmagnola CS hemp EOs

Table S1. Chemical composition of Futura 75 and Carmagnola CS hemp EOs.

No.	Component[a]	RI[b]	RI lit.[c]	Relative peak area (%)		ID[d]
				Futura 75	Carmagnola CS	
1	α-pinene	932	932	3.93	6.44	Std
2	camphene	947	946		0.05	Std
3	β-pinene	975	974	0.85	1.91	Std
4	myrcene	991	988	1.34	7.69	Std
5	α-terpinene	1016	1014		0.05	Std
6	*p*-cymene	1024	1020		0.06	Std
7	limonene	1028	1024	0.35	2.98	Std
8	1,8-cineole	1030	1026		0.77	Std
9	(*Z*)-β-ocimene	1039	1032	0.03	0.04	Std
10	(*E*)-β-ocimene	1049	1044	0.20	0.41	Std
11	γ-terpinene	1058	1054		0.12	Std
12	terpinolene	1088	1086	0.37	2.35	Std
13	borneol	1164	1165	0.07	0.07	Std
14	α-terpineol	1189	1186		0.26	Std
15	(*Z*)-caryophyllene	1406	1408	0.41	0.34	RI,MS
16	α-*cis*-bergamotene	1415	1411	0.11		RI,MS
17	(*E*)-caryophyllene	1419	1417	33.41	35.65	Std
18	α-*trans*-bergamotene	1436	1432	2.41	0.09	RI,MS
19	α-humulene	1454	1452	10.50	13.22	Std
20	(*E*)-β-farnesene	1458	1454	2.05	0.11	Std
21	*allo*-aromadendrene	1461	1458	1.61	0.69	RI,MS
22	γ-muurolene	1484	1478	0.37	0.30	RI,MS
23	β-selinene	1486	1489	2.81	1.81	RI,MS
24	valencene	1493	1496	0.15	0.18	RI,MS
25	α-selinene	1495	1498	1.66	1.34	RI,MS
26	β-bisabolene	1509	1505	0.07		RI,MS
27	(*E,E*)-α-farnesene	1509	1505		0.17	RI,MS
28	δ-cadinene	1524	1522	0.15	0.06	RI,MS
29	selina-4(15),7(11)-diene	1539	1544	0.28	0.93	RI,MS

30	selina-3,7(11)-diene	1542	1538	1.08	0.45	RI,MS
31	(*E*)-nerolidol	1564	1561	0.22	0.27	RI,MS
32	caryophyllene oxide	1583	1583	11.24	8.15	Std
33	humulene epoxide II	1610	1608	3.35	2.37	RI,MS
34	caryophylla-4(12),8(13)-dien-5-ol	1637	1639	3.02	1.14	RI,MS
35	selina-3,11-dien-6-α-ol	1658	1642	3.08	0.69	RI,MS
36	caryophyllene-14-hydroxy-9-*epi*-(*E*)	1671	1668	2.00	0.66	RI,MS
37	α-bisabolol	1684	1685	0.11	0.05	Std
38	cannabidiol	2427	2430	6.46	4.23	Std
39	cannabichromene	2435	2440	0.13	0.10	RI,MS
	Total identified (%)			93.81	96.19	

[a] Order of elution by an HP-5MS column (30 m x 0.25 mm, 0.1 μm).

[b] Linear retention index according to Van den Dool and Kratz (1963).

[c] RI from ADAMS and/or NIST 17 and FFNSC3 libraries.

[d] Identification method: Std, comparison with analytical standard; RI, coherence of the calculated RI with those found in ADAMS, NIST 17, and FFNSC3 libraries; MS, mass spectrum overlapping with those reported in ADAMS, NIST 17, WILEY 275, and FFNSC3 libraries.

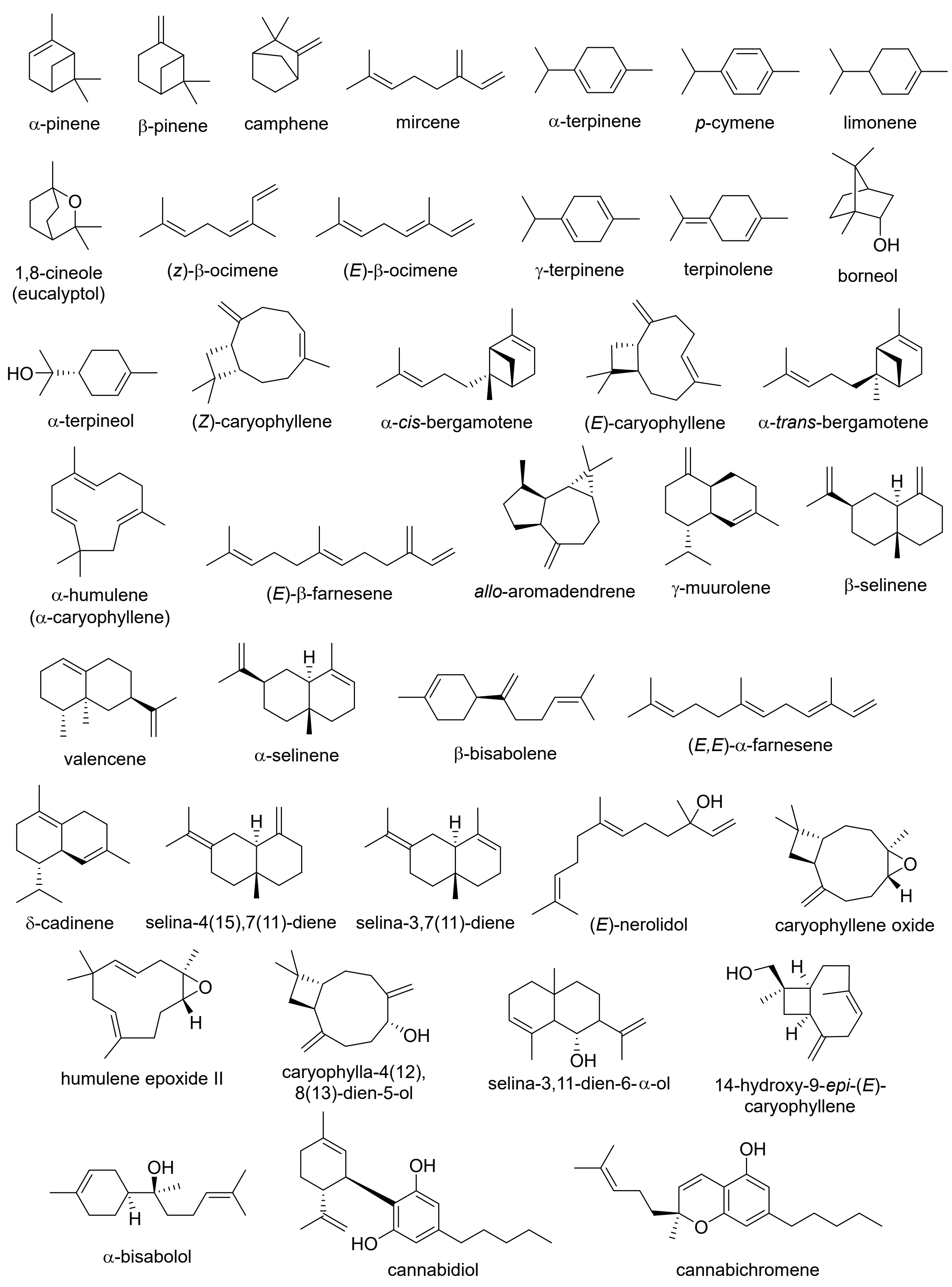

Figure S1. Chemical structures of all the 39 compounds identified in Futura 75 and Carmagnola CS hemp EOs.

Additional figures for FT-IR spectra

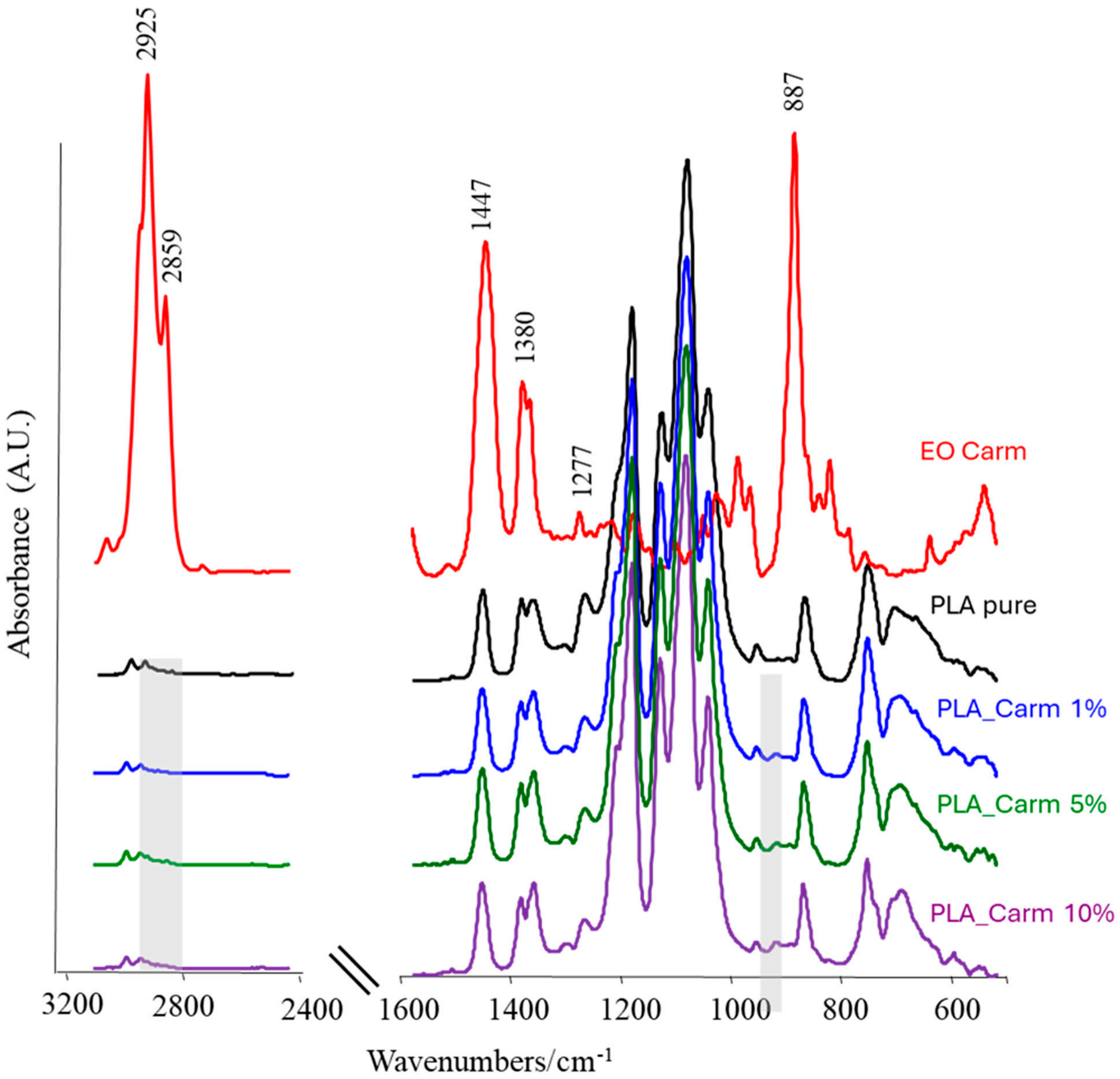

Figure S2. Selected zones of IR spectra of PLA_Carmagnola CS-based systems compared to pure PLA and Carmagnola EO spectra (see main text for the discussion).

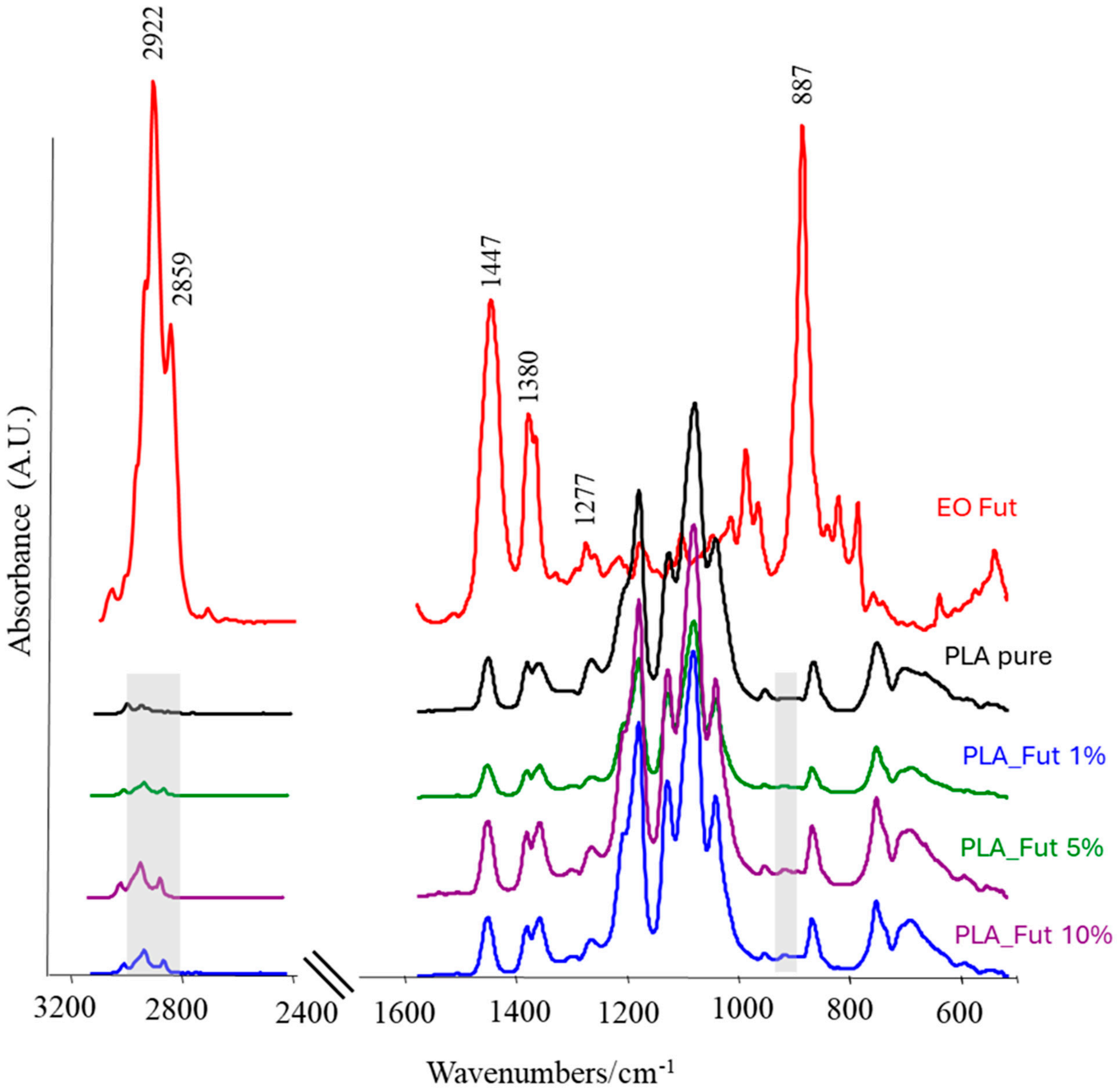

Figure S3. Selected zones of IR spectra of PLA_Futura 75-based systems compared to pure PLA and Futura 75 spectra (see main text for the discussion).

Calibration curves for Carmagnola CS and Futura 75 in water/ethanol 1:4, v/v

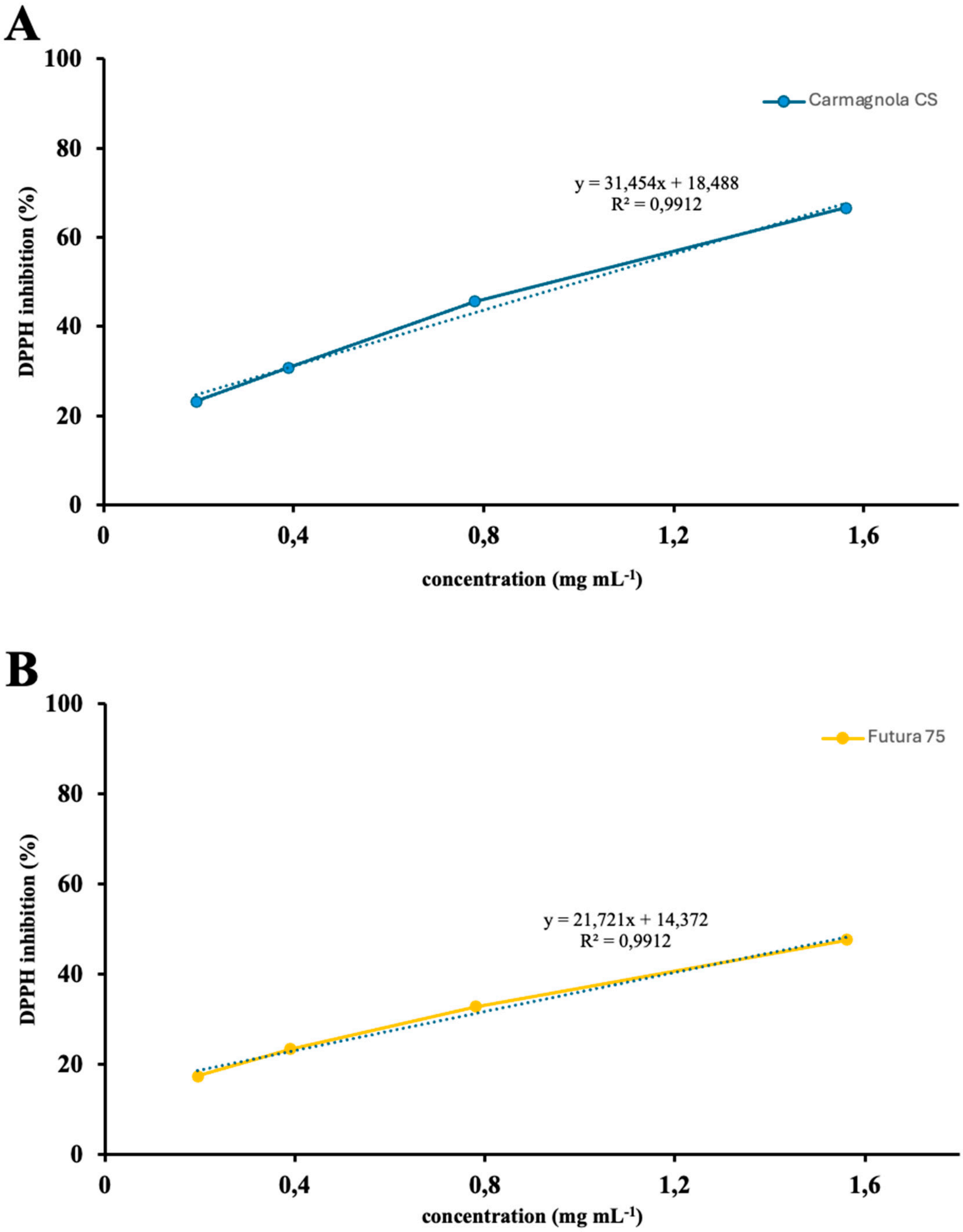

Figure S4. Calibration curves relative to DPPH inhibition of different concentrations of EOs (**A**, Carmagnola CS; **B**, Futura 75) in water/ethanol 1:4, v/v.
